# Erythropoietic responses to a series of repeated maximal dynamic and static apnoeas in elite and non-breath-hold divers

**DOI:** 10.1007/s00421-019-04235-1

**Published:** 2019-09-28

**Authors:** Antonis Elia, Matthew J. Barlow, Kevin Deighton, Oliver J. Wilson, John P. O’Hara

**Affiliations:** 1 Research Institute for Sport, Physical Activity and Leisure, Leeds Beckett University, Leeds, UK; 2 Division of Environmental Physiology, School of Chemistry, Bioengineering and Health, KTH Royal Institute of Technology, Stockholm, Sweden

**Keywords:** Erythropoietin, Hypoxia, Breath-hold, Desaturation, Diving response, Erythropoiesis

## Abstract

**Purpose:**

Serum erythropoietin (EPO) concentration is increased following static apnoea-induced hypoxia. However, the acute erythropoietic responses to a series of dynamic apnoeas in non-divers (ND) or elite breath-hold divers (EBHD) are unknown.

**Methods:**

Participants were stratified into EBHD (*n* = 8), ND (*n* = 10) and control (*n* = 8) groups. On two separate occasions, EBHD and ND performed a series of five maximal dynamic apnoeas (DYN) or two sets of five maximal static apnoeas (STA). Control performed a static eupnoeic (STE) protocol to control against any effects of water immersion and diurnal variation on EPO. Peripheral oxygen saturation (SpO_2_) levels were monitored up to 30 s post each maximal effort. Blood samples were collected at 30, 90, and 180 min after each protocol for EPO, haemoglobin and haematocrit concentrations.

**Results:**

No between group differences were observed at baseline (*p* > 0.05). For EBHD and ND, mean end-apnoea SpO_2_ was lower in DYN (EBHD, 62 ± 10%, *p* = 0.024; ND, 85 ± 6%; *p* = 0.020) than STA (EBHD, 76 ± 7%; ND, 96 ± 1%) and control (98 ± 1%) protocols. EBHD attained lower end-apnoeic SpO_2_ during DYN and STA than ND (*p* < 0.001). Serum EPO increased from baseline following the DYN protocol in EBHD only (EBHD, *p* < 0.001; ND, *p* = 0.622). EBHD EPO increased from baseline (6.85 ± 0.9mlU/mL) by 60% at 30 min (10.82 ± 2.5mlU/mL, *p* = 0.017) and 63% at 180 min (10.87 ± 2.1mlU/mL, *p* = 0.024). Serum EPO did not change after the STA (EBHD, *p* = 0.534; ND, *p* = 0.850) and STE (*p* = 0.056) protocols. There was a significant negative correlation (*r* = − 0.49, *p* = 0.003) between end-apnoeic SpO_2_ and peak post-apnoeic serum EPO concentrations.

**Conclusions:**

The novel findings demonstrate that circulating EPO is only increased after DYN in EBHD. This may relate to the greater hypoxemia achieved by EBHD during the DYN.

## Introduction

Hypoxia is a condition of reduced oxygen concentration in breathable air or blood. Elite breath-hold divers (EBHD) regularly endure periods of acute hypoxemia (oxygen saturation levels < 90%) during their training sessions, interspaced by periods of normal breathing (1–2 min). Breath-holding (apnoea) triggers a series of physiological modifications known as the diving reflex which collectively lower oxygen utilisation and in turn, prolong apnoeic durations. The diving reflex is characterised by an initial parasympathetically-induced bradycardial response (Schagatay and Holm 1996), followed by a sympathetically-induced peripheral vasoconstriction of non-vital organs and extremities (Campbell et al. 1969), with oxygenated blood preferentially redistributed to the vital organs (Sterba and Lundgren 1988).

During apnoeic periods, systemic hypoxemia is induced in renal vascular beds (Bron et al. 1966). This stimulates the release of the glycoprotein hormone erythropoietin (EPO) from the renal peritubular fibroblasts into the circulation (Elliott 2008; Jelkmann 2011). The magnitude of EPO release is directly proportional to the level of hypoxia (Eckardt et al. 1989; Knaupp et al. 1992), and the transcription of EPO is controlled, at the cellular level, by hypoxia-inducible transcription factors (Wang and Semenza 1993). EPO is responsible for activating the proliferation and maturation of red blood cells and haemoglobin (Eckardt et al. 1989; Jelkmann 1992; Lundby et al. 2007; Elliot 2008). Higher resting haemoglobin concentrations have been documented in breath-hold divers compared with untrained individuals (Richardson et al. 2005; Fernandez et al. 2017). It is possible that these differences may be influenced by the level of EPO hormone secreted by the kidneys into the systemic circulation in response to intermittent hypoxia, causing differentiation of the precursors that become haemoglobin-containing red blood cells.

To date, only two studies have investigated the erythropoietic effect of apnoea-induced hypoxia (de Bruijn et al. 2008; Kjeld et al. 2015). De Bruijn et al. (2008) first reported that a series of 15 maximal dry static apnoeas performed by a group of non-divers (ND) induced acute increases in serum EPO with peak values observed within 3 h (16% increase) of the last hypoxic bout and being restored to baseline 5 h post. More recently, Kjeld et al. (2015) reported significant increases in EPO (17%) 3 h after a single bout of a combined maximal static and dynamic apnoea attempt in a group of elite breath-hold divers (EBHD). However, a distinction between the individual physiological responses to static and dynamic apnoeas was not determined. Static and dynamic apnoeas are two fundamentally different disciplines. Although both require individuals to hold their breath during the time course of their maximal attempt, the physiological demands imposed differ substantially. Indeed, Overgaard et al. (2006) reported a greater heart rate and end-tidal carbon dioxide and lower end-tidal oxygen after dynamic apnoeas when compared to dry static apnoeas, despite the ~ 75% shorter apnoeic time period in the dynamic apnoeas. The addition of contractile activity during the state of dynamic apnoea imposes a significant challenge to the diving reflex where myocardial and skeletal muscle oxygen consumption is increased, and blood flow is redistributed to meet the competing needs of both the vital organs and recruited striated muscle. Therefore, it is tempting to speculate that the nature of dynamic apnoeas (i.e. apnoea and exercise) may induce a greater hypoxemic stress and consequently, upregulate the release of EPO.

Accordingly, this study aimed to make the first investigations into the individual erythropoietic effects of static and dynamic apnoeas performed by EBHD and ND. We hypothesise that the greater hypoxemia associated with dynamic apnoeas will stimulate greater EPO concentration compared with static apnoeas.

## Materials and methods

### Participants

Twenty-six male participants volunteered for this study and were differentiated into three groups including, EBHD, ND and control. EBHD had 6 ± 2 years of apnoea practice and their training regime consisted of 9 ± 1 h per week of apnoea-related activities (Table 1). All breath-hold divers were national team members, of which four were current and two former national record holders (Table 1). The ND were physically active individuals and had no prior breath-hold diving experience. The control group consisted of eight physically active individuals of which two of them were recruited from the ND group. Participants were healthy, non-smoking, habitual sea-level residents and provided written informed consent before the study. All experimental procedures were completed in accordance with declaration of Helsinki and institutional ethical approval.

**Table 1:** Mean (± SD) participant characteristics

Variables	EBHD (*n=* 8)	ND (*n=* 10)	Control (*n=* 8)
Height (m)	1.83 ± 0.05	1.82 ± 0.06	1.78 ± 0.09
Body mass (kg)	84 ± 12	85 ± 7	82 ± 11
Static apnoea (s)	376 ± 39	–	–
Dynamic apnoea without fins (m)	131 ± 41	–	–
Dynamic apnoea with fins (m)	193 ± 42	–	–

### Experimental protocol

Participants reported to the laboratory after a 12 h fast and abstinence from caffeine and alcohol containing beverages. In addition, participants were instructed to refrain from physical activity and apnoea-related activities for 24 h prior to and during the testing day. All resting data were collected during a single laboratory visit.

Following arrival at the laboratory (~25 °C), participants’ anthropometric measurements were assessed, including height and body mass (Seca, Vogel & Halke, Hamburg, Germany) (Table 1).

Participants then underwent a 20-min supine resting period. Subsequently, resting peripheral oxygen saturation (SpO_2_) was assessed using a finger pulse oximeter placed on the left-hand index finger (Nellcor PM10N, Medtronic, MN, USA) followed by two whole blood samples being drawn from a suitable vein in the antecubital fossa of the participant’s arm (median cubital vein and basilica vein) to assess serum EPO (6 mL; BD Vacutainer, 367954, Plymouth, UK), haemoglobin and haematocrit concentrations (4 mL; BD Vacutainer, K2E EDTA, BD, Plymouth, UK).

### Familiarisation session

Within 24 h of completing the baseline measurements, participants reported at the swimming facilities and a familiarisation session was performed. Participants were introduced to the static apnoea position (seated position immersed up to the neck) and the dynamic apnoea technique (horizontal underwater breast stroke swimming) and were familiarised to the trial conditions and requirements.

### Static apnoea protocol

Within a week from completing the familiarisation session, participants reported at the swimming pool (~ 28 °C) premises as during the familiarisation visit. The static apnoea protocol consisted of the participants performing two sets of five maximal static apnoeas. The two sets were separated by a 10-min seated rest and each apnoea was separated by a 2-min resting period.

Participants were instructed to hold their breath after a deep but not maximal inspiration, without prior hyperventilation or glossopharyngeal pistoning. A 1-min warning was provided prior to commencing each apnoea, participants received a nose clip 30 s prior to the apnoea to reduce any oxygen or water inspiration or oxygen loss, and a 10 s countdown was provided prior to immersing their face underwater and commencing their maximal apnoeic attempt. During each breath hold, participants received verbal information and a physical signal (gentle tap on the shoulder) every 30 s. After each breath hold, participants underwent a 2-min resting period during which they were allowed to relax and breathe normally in a seated position, whilst remaining immersed in water up to the waist. This procedure was repeated five times per set with the apnoeic duration being recorded during each maximal attempt.

The participant’s SpO_2_ (Nellcor PM10N, Medtronic, MN, USA) was recorded at 10 s intervals until 30 s after the termination of their breath-hold (Fagoni et al. 2017).

### Dynamic apnoea protocol

Within a week of completing the static apnoea protocol, participants reported at the swimming pool as in during the static apnoea protocol. The dynamic apnoea protocol consisted of performing five maximal dynamic apnoeas without fins, with each apnoeic repetition being separated by a 2-min seated rest (immersed in water up to the waist). The pre-apnoeic breathing protocol and data collection was replicated as in the static apnoea protocol with the exception that SpO_2_ was not measured during the maximal attempt but up to 30 s post the termination of each maximal attempt, due to practical implications. During each maximal dynamic apnoeic attempt the duration and distance covered was recorded.

### Control protocol

To control against any possible effects of whole-body immersion in water and diurnal variation in serum EPO concentration, a control group performed a static eupnoeic (normal breathing) protocol. The static eupnoeic protocol replicated the water exposure times, resting periods and data collection time points of the static apnoea protocol and replaced apnoeas with normal breathing periods. The static eupnoeic protocol was based on the static apnoeic protocol as the water exposure periods were longer in the static compared with the dynamic apnoea protocol.

Participants reported to the swimming pool facilities as during the apnoea measurements, at the same time period and were immersed in water up to the neck.

### Post-apnoea blood sample

At completion of the apnoeic and control protocols, a cannula was inserted into a suitable median cubital or basilic vein of the participant’s arm and two blood samples were drawn at 30, 90 and 180 min after the last apnoeic/eupnoeic repetition to determine the level of circulating EPO (6 mL; BD Vacutainer, 367954, Plymouth, UK), haemoglobin and haematocrit (4 mL; BD Vacutainer, K2E EDTA, BD, Plymouth, UK).

### Blood sample treatment and analysis

Samples for serum EPO were gently inverted, allowed to coagulate at room temperature for 20 min, and centrifuged (ALC Multispeed Refrigerated centrifuge, PK131R, London, United Kingdom) at 4000 rpm for 10 min at 4 °C. Samples were then aliquoted into 1.5 mL eppendorf tubes and stored at − 80 °C until an enzyme-linked immunosorbent assay analysis was performed (R&D systems, Quantikine IVD ELISA, Human Erythropoietin, DEP00, sensitivity 0.6 mIU/mL; intra-assay variability ~ 3.0%). For haemoglobin and haematocrit concentrations, samples were gently inverted for the EDTA to bind to calcium ions thus blocking the coagulation cascade and were analysed within 6 h of collection (Advia 2120i Haematology System, Siemens Healthcare, Surrey, UK; intra-assay variability ~ 1%).

### Changes in plasma volume, blood volume and red cell volume

Plasma, blood and red cell volume changes for each post-apnoeic time point were determined using the methods of Dill and Costill (1974).

### Statistical analysis

All participants completed the protocols successfully and all data were statistically analysed using the IBM SPSS statistics software version 21. The Shapiro–Wilk test was used to assess normality, whereas homogeneity was assessed using Levene’s test. Sphericity was assessed using Mauchly’s test of sphericity; where the assumption of sphericity was violated, the Greenhouse–Geisser correction was applied. Repeated measures ANOVA with post hoc contrast comparisons were used to assess differences between and within groups for baseline measurements and other collection time points for SpO_2_, serum EPO, haemoglobin and haematocrit concentrations. MANOVAS were used to assess differences in collection time points between groups (EBHD vs ND) and conditions (Static vs Dynamics vs Control). Pearson correlation was used to assess the relationship between end-apnoeic SpO_2_ and peak serum EPO concentrations, and to examine the relationship between post-apnoeic erythropoietin concentrations and plasma volume, blood volume and red cell volume. Where appropriate, effect size, partial eta squared (*η*^2^) and power (*β*) are also presented. Data are reported as means ± SD and significance was accepted at *p* < 0.05, and *p* = 0.000 was reported as *p* < 0.001. GraphPad Prism version 7.0c was used to construct figures.

## Results

### Control

Mean SpO_2_ was not significantly different from baseline (98 ± 1%) during the static eupnoeic protocol (98 ± 1%) (*p* = 1). There was a trend for serum EPO concentrations to gradually decrease from baseline concentrations (8.27 ± 3.63 mlU/mL) to 6.66 ± 1.55 mlU/mL (30 min), 5.95 ± 1.64 mlU/mL (90 min) and 5.34 ± 0.90 mlU/mL (180 min) post the completion of the static eupneic protocol (*p* = 0.056, partial *η*^2^ = 0.358).

### Static apnoeas

Mean static apnoea duration was on average 67 ± 3% longer (*p* < 0.001, partial *η*^2^ = 0.744, *β* = 1) during each successive apnoeic attempt in EBHD than ND, with a mean duration of 218 ± 21 s (range from 130 to 350 s) in EBHD compared with 74 ± 7 s (range from 30 to 183 s) in ND, respectively.

### Dynamic apnoeas

There was a between-group difference in distance covered during dynamic apnoeas (*p* < 0.001, partial *η*^2^ = 0.751, *β* = 1). The distance covered was 66 ± 4% longer in EBHD than ND during all apnoeic attempts with a mean distance covered of 66 ± 5 m (range from 46 to 126 m) compared with 22 ± 1 m (range from 14 to 37 m) in ND. Mean absolute apnoeic duration was significantly (*p* < 0.001, partial *η*^2^ = 0.641, *β* = 1) longer in EBHD (EBHD 94 ± 22 s) than ND (ND 42 ± 13 s).

### Peripheral oxygen saturation

Mean SpO_2_ was significantly different from baseline during the static apnoea repetitions in EBHD (*p* < 0.001, partial *η*^2^ = 0.638, *β* = 0.992), but not in ND (*p* = 0.327, partial *η*^2^ = 0.131, *β* = 0.248). EBHD attained significantly lower SpO_2_ (*p* = 0.001, partial *η*^2^ = 0.558, *β* = 0.982) during each successive apnoeic repetition with a mean end-apnoeic SpO_2_ of 76 ± 5% compared to 96 ± 1% in ND. The dynamic apnoea protocol induced a significant decrease in mean SpO_2_ from baseline in both groups (EBHD, *p* < 0.001, partial *η*^2^ = 0.775, *β* = 1; ND, *p* < 0.001, partial *η*^2^ = 0.685, *β* = 1). EBHD reached significantly lower SpO_2_ (mean end-apnoeic SpO_2_ 62 ± 10%) at all apnoeic repetitions when compared to ND (mean end-apnoeic SpO_2_ 85 ± 6%) (*p* < 0.001, partial *η*^2^ = 0.693, *β* = 1). When the end-apnoeic SpO_2_ for each group was compared between protocols (static vs dynamic), significantly lower SpO_2_ were attained for both groups during the dynamic apnoea protocol (EBHD, *p* = 0.004, partial *η*^2^ = 0.456, *β* = 0.889; ND, *p* < 0.001, partial *η*^2^ = 0.566, *β* = 0.99). Significantly lower SpO_2_ levels were attained for both groups during dynamic apnoeas (*p* < 0.0001) versus the control protocol, whereas only the EBHD group reached significantly lower SpO_2_ levels during the static apnoea protocol versus the control protocol (EBHD, *p* < 0.0001, partial *η*^2^ = 0.467, *β* = 0.996; ND, *p* = 0.366, partial *η*^2^ = 0.066, *β* = 0.242).

### Erythropoietin

**Fig. 1:**
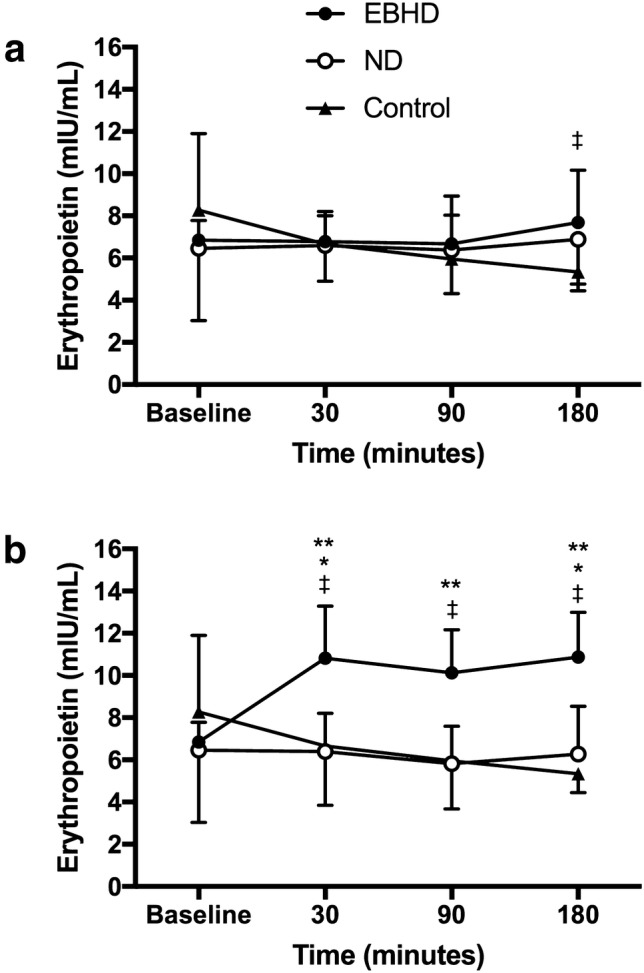
Change in mean EPO (mlU/mL) concentrations from baseline to 180 min post-apnoeas. Data are presented as mean ± SD. Significant difference (*p<* 0.05) compared to baseline is denoted as asterisk, between dynamic and static apnoea protocols is denoted as double asterisk, between dynamics and control protocols is denoted as dagger. **a** Static apnoea and control protocols. **b** Dynamic apnoea and control protocols. *EBHD* elite breath-hold divers, *ND* non-divers

**Fig. 2:**
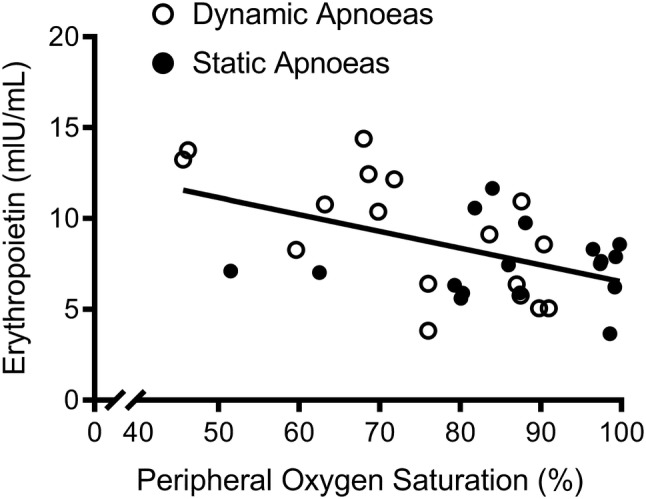
Relationship between end-apnoeic peripheral oxygen saturation levels and peak post-apnoeic serum erythropoietin concentrations for both groups and apnoeic protocols

Mean post-apnoeic EPO concentrations were not different from baseline during the static apnoea protocol for either groups (EBHD, *p* = 0.534, partial *η*^2^ = 0.097, *β* = 0.183; ND, *p* = 0.850, partial *η*^2^ = 0.006, *β* = 0.055) or when compared between groups (*p* = 0.471, partial *η*^2^ = 0.033, *β* = 0.107) (Fig. 2). Mean post-apnoeic EPO concentration was significantly different from baseline during the dynamic apnoea protocol in EBHD (*p* < 0.001, partial *η*^2^ = 0.584, *β* = 0.992) but not in ND (*p* = 0.622, partial *η*^2^ = 0.062, *β* = 0.157) (Fig. 1).

Specifically, serum EPO concentration was 60% higher than baseline (6.85 ± 0.9 mlU/mL) at 30 min (10.82 ± 2.5 mlU/mL, *p* = 0.017) and 63% higher at 180 min post-dynamic apnoeas (10.87 ± 2.1 mlU/mL, *p* = 0.024) in EBHD. There was a trend for increased EPO at 90 min post-apnoea (10.13 ± 2.0 mlU/mL, *p* = 0.058). In the EBHD group, there was inter-individual variability in the time to peak serum EPO concentration in response to dynamic apnoeas. Mean peak serum EPO concentration was 78 ± 43% (45–151%) higher than baseline after dynamic apnoeas (*p* = 0.001) in the EBHD. When the mean post-apnoeic EPO concentrations were compared between groups, the EBHD attained significantly higher EPO concentrations during all timepoints  when compared with the ND group (*p* = 0.002, partial *η*^2^ = 0.475, *β* = 0.946) (Fig. 1).

EPO concentrations were significantly higher in response to the dynamic versus static apnoea protocol in EBHD (*p* = 0.001, partial *η*^2^ = 0.548, *β* = 0.969) (Fig. 2), but EPO concentrations were not different between protocols in ND (*p* = 0.867, partial *η*^2^ = 0.002, *β* = 0.053). Additionally, EPO concentrations were significantly higher in response to the apnoeic protocols versus the control protocol in EBHD (Dynamics, *p* = 0.001, partial *η*^2^ = 0.548, *β* = 0.969; Statics, *p* = 0.043, partial *η*^2^ = 0.196, *β* = 0.595), whereas no differences were reported between protocols in ND (Dynamics, *p* = 0.066, partial *η*^2^ = 0.117, *β* = 0.484; Statics, *p* = 0.152, partial *η*^2^ = 0.117, *β* = 0.341) (Fig. 1).

There was a significant (*p* = 0.003) moderate negative correlation (*r* = − 0.49) between end-apnoeic SpO_2_ and peak post-apnoeic serum EPO concentrations (Fig. 2).

### Haemoglobin and haematocrit

Mean post-apnoeic haemoglobin and haematocrit concentrations were not different from baseline during the static (haemoglobin, *p* = 0.136, haematocrit, *p* = 0.064) or dynamic (haemoglobin, *p* = 0.427, haematocrit, *p* = 0.522) apnoea protocol for either groups (Table 2).

**Table 2:** Change in mean haemoglobin (g/dl) and haematocrit (%) (mlU/mL) concentrations from baseline to 180 min post-apnoeas

Protocol	Haemoglobin (g/dl)				Haematocrit (%)			
	Baseline	30	90	180	Baseline	30	90	180
EBHD								
A	15 ± 0.60	14.54 ± 0.99	14.73 ± 1.05	15.01 ± 0.57	44 ± 1.62	42 ± 2.36	42 ± 2.77	42 ± 2.09
B		14.96 ± 0.77	15.37 ± 1.79	15.17 ± 2.03		43 ± 2.62	42 ± 5.50	43 ± 6.06
ND								
A	14.9 ± 0.44	14.81 ± 0.56	14.68 ± 0.57	14.67 ± 0.51	45 ± 1.76	44 ± 2	44 ± 1.66	44 ± 1.32
B		14.83 ± 0.74	14.57 ± 0.91	14.71 ± 0.86		45 ± 3.30	44 ± 4.08	44 ± 4.42

Data are presented as mean ± SD

*A* Static apnoea, *B* Dynamic apnoea, *EBHD* elite breath-hold divers, *ND* non-divers

### Plasma volume, blood volume and red cell volume

**Fig. 3:**
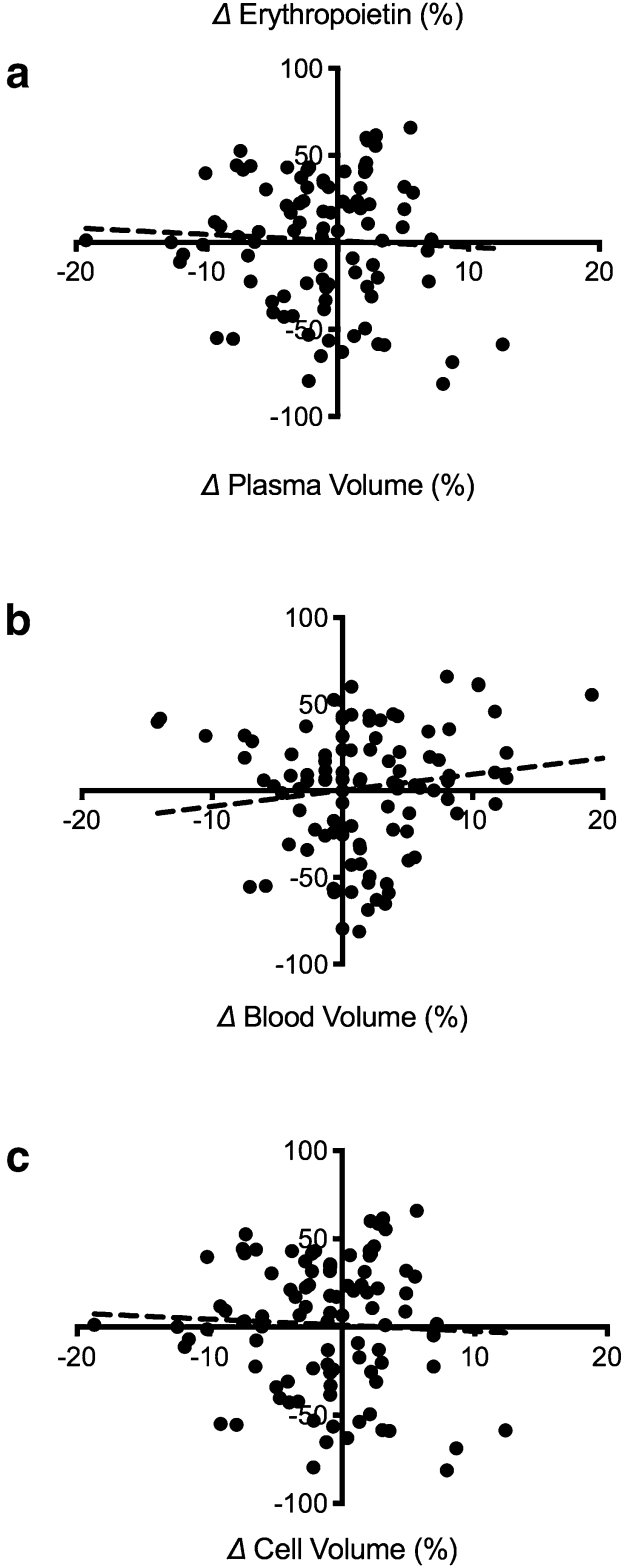
Relationship between post-apnoeic delta percentage change for erythropoietin concentrations and plasma volume (**a**), blood volume (**b**) and cell volume (**c**) for both groups and apnoeic protocols

Plasma volume, blood volume or red cell volume did not change for either protocol or group (*p* = 0.83). There was no relationship between post-apnoeic delta percentage change in EPO concentration and delta percentage change in plasma volume (*r* = − 0.052, *p* = 0.613), blood volume (*r* = 0.151, *p* = 0.122) or red cell volume (*r* = − 0.048, *p* = 0.643) (Fig. 3).

## Discussion

This is the first study to make a novel distinction between the acute effects of static and dynamic apnoeas on erythropoietic responses in ND and EBHD. The primary findings were that: (1) dynamic apnoeas induced a more pronounced oxygen desaturation rate compared with static apnoeas, (2) which led into a significant increase in circulating EPO in EBHD only, (3) with no effect of static or eupnoea protocols on EPO. These findings confirm our hypothesis that a series of maximal dynamic apnoeas can elicit a greater hypoxemia than a series of maximal static apnoeas, and that the hypoxemia achieved during dynamic apnoeas is associated with a robust increase in circulating EPO.

Dynamic apnoeas induced a greater reduction in SpO_2_ than static apnoeas in both groups, despite significantly lower absolute apnoeic durations during the dynamic apnoea protocol compared with static apnoeas. These data suggest that tissue oxygen consumption was greater during repeated maximal dynamic apnoeas compared with repeated maximal static apnoeas, signifying that the addition of contractile activity during the state of apnoea imposes a greater hypoxemic stress. During both apnoeic protocols EBHD achieved significantly longer apnoeic durations and reached lower end-apnoeic SpO_2_ than ND. More experienced breath-hold divers, such as those recruited in the current study, are able to withstand the physiological breaking point, supress the urge to breathe and sustain prolonged apnoeic durations. As a consequence, EBHD are exposed to greater hypoxemic conditions during maximal apnoeic attempts than ND (Joulia et al. 2002). Evidence suggests the response of EBHD to apnoeas is trainable as Joulia et al. (2003) demonstrated that 3 months of apnoeic training significantly delayed the physiological breaking point, improved hypoxic tolerance and prolonged apnoeic durations.

To the best of our knowledge, this is the first study to report the erythropoietic responses to a series of maximal dynamic apnoeas performed by EBHD and ND. Increases in serum EPO in response to the dynamic apnoea protocol occurred only in the EBHD group, with no effect of the static apnoea or eupnoea protocol on EPO (Fig. 1). Additionally, there was no relationship between post-apnoeic EPO concentrations and blood volume, red cell volume or plasma volume (Fig. 3). Collectively, this suggests that the significant increases in serum EPO that occurred only in EBHD after completing the dynamic apnoea protocol were likely caused by the greater dynamic apnoea-induced hypoxia compared with ND and not by a circadian rhythm of EPO production, water immersion or due to haemoconcentration (Cahan et al. 1992; Klausen et al. 1996). In addition, in line with the literature, we identified for both groups (EBHD and ND) a moderate negative correlation between end-apnoeic SpO_2_ and peak post-apnoeic EPO concentrations (Fig. 2). Therefore, the lower SpO_2_ attained by the EBHD compared with ND during the dynamic apnoea protocol may have elicited a greater hypoxic stimulus for EPO release.

Despite the relative short (< 10 min), intermittent nature of the hypoxic exposures utilised in the current study, the observed serum EPO increases reported 3 h post (4.02 mlU/mL; 63% increase) a series of only five maximal dynamic apnoeas in EBHD are greater than those previously reported by de Bruijn et al. (2008) (1.38 mlU/mL; 16% increase) following a series of 15 maximal dry static apnoeas and by Kjeld et al. (2015) (1.8 mlU/mL; 17% increase) post a series of a combined maximal static and dynamic apnoea. The higher serum EPO concentrations observed in the present study, despite only one third of the number of apnoeas compared with de Bruijn et al. (2008), are probably attributed to the lower SpO_2_ levels attained by our EBHD group as a result of the longer apnoeic durations achieved. Additionally, the EBHD individual maximum serum EPO concentrations post the dynamic apnoea protocol are higher than those reported by Klausen et al. (1996) following 2 h of pokilocapnic hypoxia (28%, 8 ± 0.84 to 10.24 ± 0.95 mlU/mL) and by Ge et al. (2002) following 6 h at a simulated altitude of 2800 m and 24 h at 1780 m and 2085 m. However, it is currently unknown whether the present increases in serum EPO after repeated dynamic apnoeas, would ultimately translate to chronic increases in red blood cell mass and haemoglobin concentrations. Accordingly, future research should aim to investigate the longitudinal effects of dynamic apnoea training.

No differences in serum EPO from baseline were observed post the static apnoea protocol for either group (Fig. 1). This is contrary to de Bruijn et al. (2008) who demonstrated a 16% (1.38 mlU/L) increase 3 h post a series of repeated dry static apnoeas in ND. Although the lack of EPO release in our ND group may be explained by their lower end-apnoeic desaturation levels attained compared with de Bruijn et al. (2008) ND group, our breath-hold divers’ end-apnoeic SpO_2_ levels are comparable to those of de Bruijn et al. (2008). However, similarly to our ND group no significant erythropoietic differences were observed in the EBHD post the static apnoea protocol. This novel observation might suggest that chronic apnoeic training (exposure to prolonged and repetitive hypoxic periods) may attenuate the decrease in renal blood flow and subsequently suppress the release of EPO. Indeed, endurance training can attenuate the reduction of renal vascular blood flow at a given absolute work rate in humans and rodents (Clausen et al. 1973; Armstrong and Laughlin 1984; DiCarlo and Bishop 1990; Musch et al. 1991; Lash et al. 1993; Yen et al. 1995; Proctor et al. 2001). In rabbits, endurance exercise reduced renal sympathetic nerve activity, partly, due to enhanced cardiac baroreflex inhibition of sympathetic outflow to the mesenteric and renal circulation (DiCarlo et al. 1997; Mueller et al. 1998; De Moraes et al. 2004). Moreover, in vitro studies examining the conduit arteries and microcirculation of the renal vasculature revealed enhanced production of and/or sensitivity to endogenous endothelial dilators (Chen et al. 1999, 2001; Chies et al. 2004; De Moraes et al. 2004; Moyna and Thompson 2004). Collectively, the insignificant erythropoietic responses in our EBHD group following the static apnoea protocol might be explained by the training-induced renal adaptations and attenuation of renal vasoconstriction. However, further research is necessary to determine the extent to which our findings are the result of a training-induced adaptation of the renal vasculature.

In conclusion, we demonstrated that repeated maximal dynamic apnoeas significantly reduced SpO_2_ compared with static apnoeas in both EBHD and ND. Hypoxemia was greatest in EBHD in response to the dynamic apnoeas and this was associated with an increase in serum EPO in EBHD only. Accordingly, future research should aim to assess the longitudinal effects of dynamic apnoea training.

## References

[CR1] Armstrong RB, Laughlin MH (1984). Exercise blood flow patterns within and among rat muscles after training. Am J Physiol.

[CR2] Bron KM, Murdaugh HV, Millen JE, Lenthall R, Raskin P, Robin ED (1966). Arterial constrictor response in a diving mammal. Science.

[CR3] Cahan C, Decker MJ, Arnold JL, Washington LH, Veldhuis JD, Goldwasser E, Strohl KP (1992). Diurnal variations in serum erythropoietin levels in healthy subjects and sleep apnea patients. J Appl Physiol.

[CR4] Campbell LB, Gooden BA, Horowitz JD (1969). Cardiovascular responses to partial and total immersion in man. J Physiol.

[CR5] Chen Y, Collins HL, DiCarlo SE (1999). Daily exercise enhances acetylcholine-induced dilation in mesenteric and hindlimb vasculature of hypertensive rats. Clin Exp Hypertens.

[CR6] Chen SJ, Wu CC, Yen MH (2001). Exercise training activates large-conductance calcium-activated K(+) channels and enhances nitric oxide production in rat mesenteric artery and thoracic aorta. J Biomed Sci.

[CR7] Chies AB, de Oliveira AM, Pereira FC, de Andrade CR, Correa FM (2004). Phenylephrine-induced vasoconstriction of the rat superior mesenteric artery is decreased after repeated swimming. J Smooth Muscle Res.

[CR8] Clausen JP, Klausen K, Rasmussen B, Trap-Jensen J (1973). Central and peripheral circulatory changes after training of the arms or legs. Am J Physiol.

[CR9] de Bruijn R, Richardson M, Schagatay E (2008). Increased erythropoietin concentration after repeated apneas in humans. Eur J Appl Physiol.

[CR10] De Moraes R, Gioseffi G, Nobrega AC, Tibirica E (2004). Effects of exercise training on the vascular reactivity of the whole kidney circulation in rabbits. J Appl Physiol.

[CR11] DiCarlo SE, Bishop VS (1990). Regional vascular resistance during exercise: role of cardiac afferents and exercise training. Am J Physiol.

[CR12] DiCarlo SE, Stahl LK, Bishop VS (1997). Daily exercise attenuates the sympathetic nerve response to exercise by enhancing cardiac afferents. Am J Physiol.

[CR13] Dill DB, Costill DL (1974). Calculation of percentage changes in volumes of blood, plasma, and red cells in dehydration. J Appl Physiol.

[CR14] Eckardt KU, Boutellier U, Kurtz A, Schopen M, Koller EA, Bauer C (1989). Rate of erythropoietin formation in humans in response to acute hypobaric hypoxia. J Appl Physiol.

[CR15] Elliott S (2008). Erythropoiesis-stimulating agents and other methods to enhance oxygen transport. Br J Pharmacol.

[CR16] Fagoni N, Taboni A, Vinetti G, Bottarelli S, Moia C, Bringard A, Ferretti G (2017). Alveolar gas composition during maximal and interrupted apnoeas in ambient air and pure oxygen. Respir Physiol Neurobiol.

[CR17] Fernández FdA, González-Ravé JM, Juárez D (2017). Breath-hold diving performance factors. J Hum Sport Ex.

[CR18] Ge RL, Witkowski S, Zhang Y (2002). Determinants of erythropoietin release in response to short-term hypobaric hypoxia. J Appl Physiol.

[CR19] Jelkmann W (1992). Erythropoietin: structure, control of production, and function. Physiol Rev.

[CR20] Jelkmann W (2011). Regulation of erythropoietin production. J Physiol.

[CR21] Joulia F, Steinberg JG, Wolff F, Gavarry O, Jammes Y (2002). Reduced oxidative stress and blood lactic acidosis in trained breath-hold human divers. Respir Physiol Neurobiol.

[CR22] Joulia F, Steinberg JG, Faucher M, Jamin T, Ulmer C, Kipson N, Jammes Y (2003). Breath-hold training of humans reduces oxidative stress and blood acidosis after static and dynamic apnea. Respir Physiol Neurobiol.

[CR23] Kjeld T, Jattu T, Nielsen HB, Goetze JP, Secher NH, Olsen NV (2015). Release of erythropoietin and neuron-specific enolase after breath holding in competing free divers. Scand J Med Sci Sports.

[CR24] Klausen T, Poulsen TD, Fogh-Andersen N, Richalet JP, Nielsen OJ, Olsen NV (1996). Diurnal variations of serum erythropoietin at sea level and altitude. Eur J Appl Physiol Occup Physiol.

[CR25] Knaupp W, Khilnani S, Sherwood J, Scharf S, Steinberg H (1992). Erythropoietin response to acute normobaric hypoxia in humans. J Appl Physiol.

[CR26] Lash JM, Reilly T, Thomas M, Bohlen HG (1993). Adrenergic and pressure-dependent vascular regulation in sedentary and trained rats. Am J Physiol.

[CR27] Lundby C, Thomsen JJ, Boushel R, Koskolou M, Warberg J, Calbet JA, Robach P (2007). Erythropoietin treatment elevates haemoglobin concentration by increasing red cell volume and depressing plasma volume. J Physiol.

[CR28] Moyna NM, Thompson PD (2004). The effect of physical activity on endothelial function in man. Acta Physiol Scand.

[CR29] Mueller PJ, O’Hagan KP, Skogg KA, Buckwalter JB, Clifford PS (1998). Renal hemodynamic responses to dynamic exercise in rabbits. J Appl Physiol.

[CR30] Musch TI, Terrell JA, Hilty MR (1991). Effects of high-intensity sprint training on skeletal muscle blood flow in rats. J Appl Physiol.

[CR31] Overgaard K, Friis S, Pedersen RB, Lykkeboe G (2006). Influence of lung volume, glossopharyngeal inhalation and P(ET) O_2_ and P(ET) CO_2_ on apnea performance in trained breath-hold divers. Eur J Appl Physiol.

[CR32] Proctor DN, Miller JD, Dietz NM, Minson CT, Joyner MJ (2001). Reduced submaximal leg blood flow after high-intensity aerobic training. J Appl Physiol.

[CR33] Richardson M, de Bruijn R, Holmberg HC, Bjorklund G, Haughey H, Schagatay E (2005). Increase of hemoglobin concentration after maximal apneas in divers, skiers, and untrained humans. Can J Appl Physiol.

[CR34] Schagatay E, Holm B (1996). Effects of water and ambient air temperatures on human diving bradycardia. Eur J Appl Physiol Occup Physiol.

[CR35] Sterba JA, Lundgren CE (1988). Breath-hold duration in man and the diving response induced by face immersion. Undersea Biomed Res.

[CR36] Wang GL, Semenza GL (1993). General involvement of hypoxia-inducible factor 1 in transcriptional response to hypoxia. Proc Natl Acad Sci USA.

[CR37] Yen MH, Yang JH, Sheu JR, Lee YM, Ding YA (1995). Chronic exercise enhances endothelium-mediated dilation in spontaneously hypertensive rats. Life Sci.

